# Validity of a New Portable Sensor to Measure Velocity-Based Resistance Training

**DOI:** 10.3390/mps8010009

**Published:** 2025-01-20

**Authors:** Alejandro Justo-Álvarez, Juan García-López, Rafael Sabido, Adrián García-Valverde

**Affiliations:** 1 Faculty of Physical Activity and Sports Sciences, Universidad de León, 24071 León, Spain; alexjusto96@gmail.com; 2 Sports Research Centre, Miguel Hernández University, 03202 Alicante, Spain; rsabido@umh.es; 3 Faculty of Health Science, International University Isabel I of Castile, 09003 Burgos, Spain; adriang.valverde@gmail.com

**Keywords:** strength training, instrumentation, mean propulsive velocity, peak velocity

## Abstract

This study evaluated the concurrent validity of the Vitruve linear encoder compared to the T-Force device for measuring mean propulsive velocity (MPV) and peak velocity (PV) during the free-weight bench press exercise. Thirteen resistance-trained men participated in three sessions, during which MPV and PV were recorded simultaneously by both devices. The data were analysed using one-way ANOVA, Pearson’s correlation, Bland–Altman analysis, and effect size calculations, with statistical significance set at *p* ≤ 0.05. The results showed discrepancies between the Vitruve and T-Force devices across different intensity levels. Specifically, the Vitruve device generally reported higher MPV and lower PV values, particularly at moderate and low intensities. Vitruve was deemed useful for MPV measurements, especially at velocities below 0.65 m/s during free-weight bench press exercises. In conclusion, the Vitruve device overestimated MPV and underestimated PV at moderate and low loads (>0.65 m·s^−1^), with the discrepancies increasing as velocity rose. It can provide valuable data for monitoring and assessing resistance training programs focused on MPV at heavier loads (<0.65 m·s^−1^). Researchers and practitioners should take these findings into account when incorporating the Vitruve into velocity-based strength training protocols.

## 1. Introduction

Velocity-based training is currently the most appropriate method to assess, monitor, and prescribe resistance training [1]. This method involves measuring the linear velocity throughout the concentric phase of a movement for various purposes. For instance, mean propulsive velocity (MPV) and peak velocity (PV) measurements can be useful for estimating fatigue across sets [2,3,4]. Additionally, MPV provides valuable information for optimizing athletes’ training programs [1,5]. Thus, the identification of a portable and cost-effective device (e.g., Vitruve) capable of precise measurements is crucial to fully utilize this information under field conditions.

Numerous studies have investigated the reliability and validity of commercial transducers across various exercises and movement executions [6,7]. In this sense, the T-force has been widely used and raised as one of the gold standards due to its validity and excellent reliability [8,9,10,11]. Other encoders, such as Speed4Lift (now Vitruve), have been the focus of conflicting conclusions regarding their validity and reliability [6,7,12,13]. This device has demonstrated good reliability at higher loads, suggesting its potential utility for 1RM estimation [13]. Hence, its use has been accepted under these conditions (i.e., heavy loads) despite limited supporting evidence [14]. Similarly to many other devices, Vitruve and Speed4Lift have primarily been evaluated within a narrow range of low velocities (0.17–0.8 m·s^−1^) commonly used for strength assessment [8,15]. In contrast, their accuracy at moderate and low loads (≥1 m·s^−1^), which are frequently utilized for training at near-optimal power [16], remains to be confirmed.

A previous study found the Vitruve device reliable for 20–90% of 1RM (CV < 8.8%) but invalid for estimating 1RM, regardless of the prediction model used [15]. Another recent study [16] investigated the validity and agreement between the Vitruve and T-Force transducers during squat and bench press exercises. The results showed that the Vitruve is valid and accurate only at velocities below 0.75 m·s^−1^ and 0.45 m·s^−1^, respectively. Furthermore, MPV measurements were more reliable than PV, suggesting that the Vitruve might not be suitable for precisely monitoring resistance training or assessing strength performance across the entire load–velocity curve. One of the aforementioned Vitruve studies was conducted during bench press exercises using a Smith machine [8], another during free-weight back squat exercises [15], and the last one involved a Smith machine for both back squat and bench press exercises [16]. Therefore, no previous study has been conducted using the most common bench press training exercise (i.e., free weights).

The main purpose of the present study was to evaluate the validity of Vitruve in a wide range of velocities during the free weight bench press exercise. Specifically, the results of MPV and PV during the propulsive phase of this exercise were compared to those obtained from the T-Force device, which was considered the “gold standard”, with special attention to those obtained at high velocities (>1 m/s).

## 2. Materials and Methods

### 2.1. Participants

Thirteen healthy resistance-trained men participated in the present study (23.4 ± 1.9 years, 1.81 ± 0.07 m, 78.8 ± 6.8 kg, and 3.7 ± 1.1 years of experience in weightlifting). Individuals were excluded from participation if they had experienced a musculoskeletal impairment or injury within the past two months or were unable to perform at maximal effort due to physical limitations that could impair their performance. This study was approved by the University research ethics committee, and all subjects read and signed an informed consent document before participating.

### 2.2. Procedures

After a familiarization session, the participants visited the weightlifting room, properly equipped at the University sport facilities, three times during a five-week longitudinal training program with three training sessions per week. Each visit, separated by seven training sessions, included a standardized warm-up and a one repetition-maximum (1RM) test for the bench press exercise. The warm-up consisted of five minutes of aerobic exercise (skipping rope), joint mobility drills, and three progressive sets of ten repetitions for triceps extension, band pull-aparts, and plyometric push-ups. During the 1RM test on the bench press, mean propulsive velocity (MPV) and peak velocity (PV) were recorded simultaneously for each repetition using two linear encoders (Vitruve and T-Force). An increment of the load was carried out according to the MPV of each attempt (see the detailed protocol below), which was categorized as high (≤0.65 m·s^−1^), moderate (<0.99 m·s^−1^) or low (≥1 m·s^−1^) intensity for subsequent analysis [17]. As the load increment was smaller at high intensity, the number of recorded repetitions was higher (i.e., 160, 62, and 65 recording at high, moderate, and low intensities, respectively). A sample size of more than 22 observations would be required for each of these three analysis groups. This estimation was performed using the ‘pwr’ package for R (version 4.4.2) [18], assuming a large effect size for one-way ANOVA (f = 0.4), a statistical power of 0.8, and a significance level of 0.05.

### 2.3. Bench Press One Repetition-Maximum Test (1RM)

**Figure 1:**
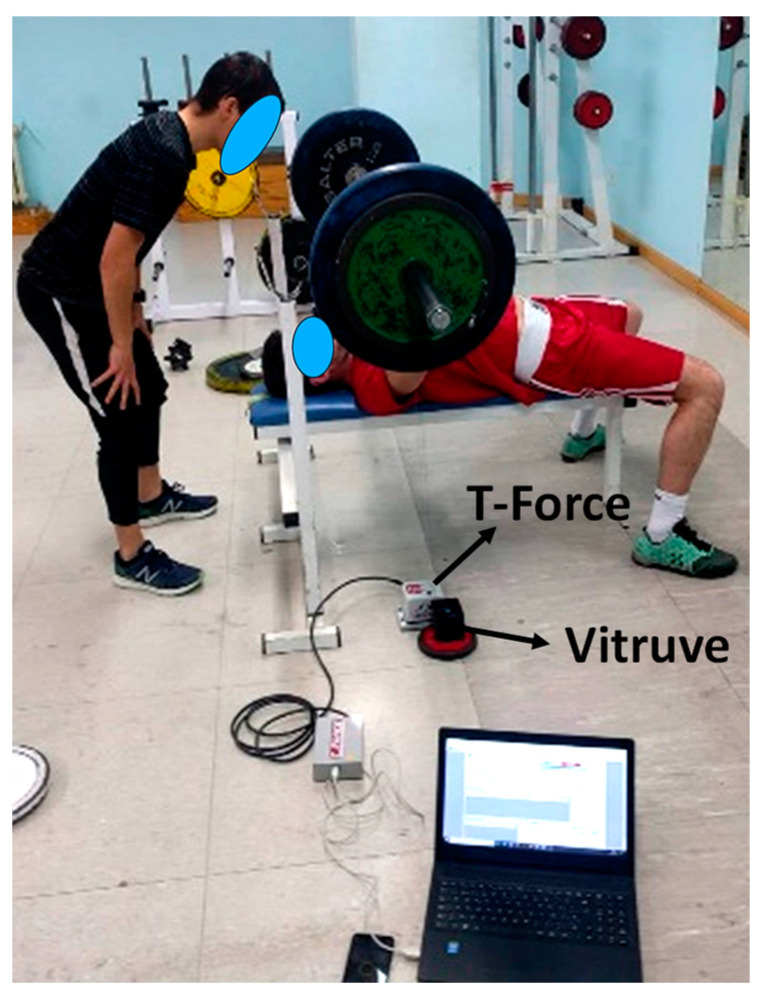
Location of the two encoders to measure the bar velocity during the bench press exercise.

Participants were instructed to lie down on a flat bench, ensuring that their head, shoulders, buttocks, and feet remained in contact with the surface throughout the press execution (Figure 1). They were required to perform an eccentric contraction until the bar lightly touched their chest, approximately 3 cm above the sternal xiphoid, followed by an isometric contraction lasting around 2 s. After this, they were instructed to perform the concentric phase of the bench press as quickly as possible.

The protocol for assessing 1RM in the bench press was previously described by González-Badillo et al. [19]. The initial load was established at 20 kg and every increment of the load was 10 kg until the attained MPV was <0.5 m·s^−1^. Then, the load was increased from 2.5 to 5 kg. The recoveries were three minutes for low and medium loads and five minutes for heavy loads. Moreover, as per the original protocol [19], the participants were required to perform three repetitions with loads capable of moving at speeds higher than 1 m·s^−1^, two repetitions with loads at speeds between 0.99 and 0.66 m·s^−1^, and just one repetition with a load moving slower than 0.65 m·s^−1^. The fastest repetition in each attempt was used for the analysis.

### 2.4. Instruments

The two encoders (Vitruve and T-Force) were arranged in parallel on the same side of the bar to avoid misalignments in between (Figure 1). Each encoder used its own software (see below) to determine the movement variables once the evaluator gave the instruction “ready” to the participant, who then started freely.

Vitruve is a linear encoder built with a nylon rope of 2 m in length and 3 mm in thickness whose weight is 365 g. The encoder has six magnets in the base to ensure a secure fix during the execution lift. It allows measurement of the linear distance of movement, mean and maximal propulsive velocity, and power values with a sample rate of 100 Hz. According to the manufacturer, it has a range measuring between 0.04 and 6 m·s^−1^. The data recorded by the Vitruve were sent to an Android phone (Xiaomi Redmi 5 Plus) through a Wi-Fi connection and analysed by specific software (Speed4Lifts App, v. 1.41).

T-Force is a linear encoder built with steelware that is 2 m in length and 0.45 mm in diameter, which can resist 5.3 N of tension. This device records a maximal velocity of 10 m·s^−1^ and maximal acceleration of 16 Gs (157 m·s^−2^). It has a sample rate of 1 kHz and an interface of conversions A/D of 14 bits of resolution. According to the manufacturer, it has an absolute error of ±1 mm and a relative error of less than 0.25%. Several authors have previously used this linear encoder and compared it with other devices [8,9,10]. Currently, it is considered the gold standard device [11]. The data recorded by the T-Force system were sent to a laptop (Lenovo B50-50 running Windows 10), where they were analysed using specific software (T-Force, v.3.70).

### 2.5. Statistical Analysis

Data analysis was performed through a one-way ANOVA to discern differences between encoders in three categorical intensities according to MPV, and the interactions were submitted to the post hoc Bonferroni test. When differences were identified, the below analyses were performed. (i) The relationship of measurements between devices was analysed with Pearson’s correlation. The criteria to interpret the strength of the r coefficients were as follows: (0.00–0.09), small (0.01–0.29), moderate (0.30–0.49), high (0.50–0.69), very high (0.70–0.89), or practically perfect (0.90–1.00) [20,21]. (ii) Bland–Altman [20,21] analysis was performed to evaluate the concurrent encoder’s validity concerning T-Force. (iii) The magnitude of differences was calculated using Hedges’ g between different devices. The g values were interpreted as trivial (g < 0.20), small (g < 0.50), moderate (g < 0.80), and large (g ≥ 0.80). Significance was set up at *p* < 0.05.

## 3. Results

Both MPV and PV were analysed. While very high correlations were observed between T-Force and Vitruve, interaction effects were found across all intensity levels. This indicates that the two devices provided differing values for the same measurements.

### 3.1. Mean Propulsive Velocity

**Figure 2:**
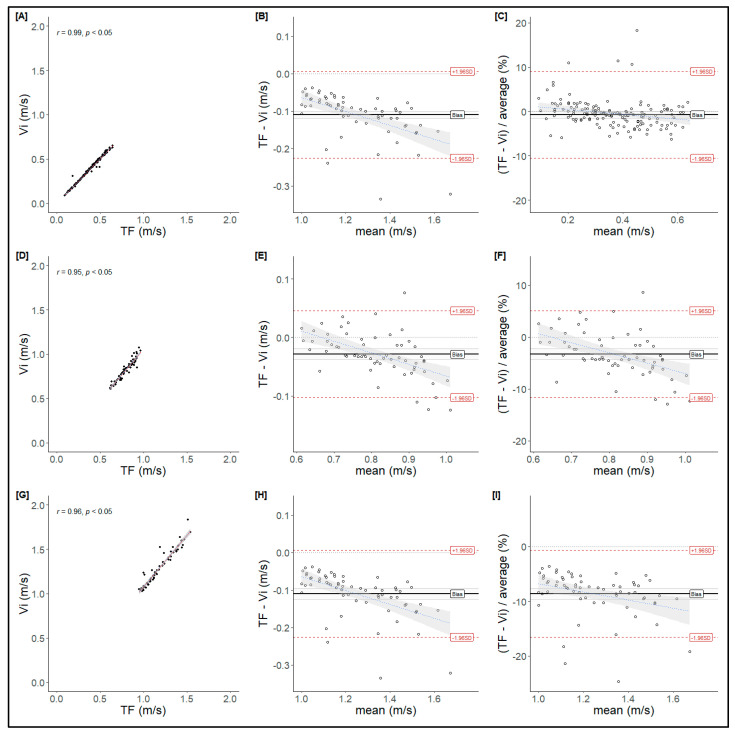
Bland–Altman analysis for mean propulsive velocity (MPV). High (**A**–**C**), moderate (**D**–**F**), and low (**G**–**I**) intensities are presented. From left to right, the regression line between hypothetical measurements from T-Force (TF) and Vitruve (Vi); plot of differences between T-Force (TF) and Vitruve (Vi) vs. mean of two measurements; and plot of differences between T-Force (TF) and Vitruve (Vi), expressed as percentages of average vs. the mean of two measurements.

MPV data from T-Force and Vitruve measurements showed an almost perfect correlation (Low: r = 0.96, *p* < 0.05; Moderate: r = 0.95, *p* < 0.05; High: r = 0.99, *p* < 0.05) (Figure 2). A Bland–Altman analysis was conducted to examine the differences between the two devices. The results indicate that Vitruve measures, on average, between 0.003 and 0.110 m·s^−1^ higher than T-Force. This difference revealed a significant systematic negative bias, particularly at moderate (Figure 2E) and low intensities (Figure 2H). Additionally, the relative error showed increased variability in the differences as the mean propulsive velocity rose (Figure 2C,F,I).

### 3.2. Peak Velocity

**Figure 3:**
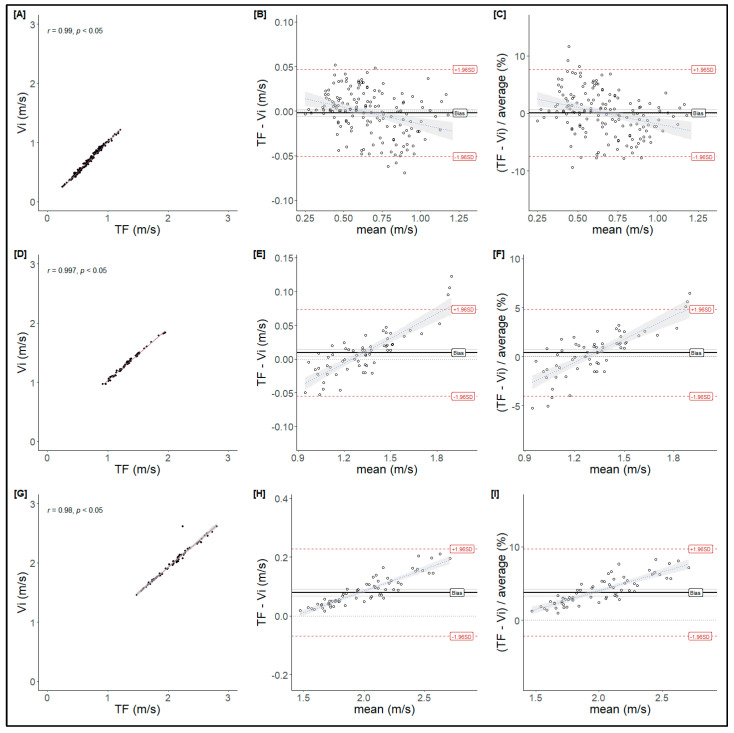
Bland–Altman analysis for peak velocity (PV). High (**A**–**C**), moderate (**D**–**F**), and low (**G**–**I**) intensities are presented. From left to right, the regression line between hypothetical measurements from T-Force (TF) and Vitruve (Vi); plot of differences between T-Force (TF) and Vitruve (Vi) vs. mean of two measurements; and plot of differences between T-Force (TF) and Vitruve (Vi), expressed as percentages of average vs. the mean of two measurements.

PV data analysis (ANOVA) showed interaction effects at moderate and low loads, indicating disagreement between T-Force and Vitruve in several measurements. Despite this, the correlation between the devices showed high values, suggesting an almost perfect relationship (Low: r = 0.98, *p* < 0.05; Moderate: r = 0.99, *p* < 0.05; High: r = 0.99, *p* < 0.05) (Figure 3). A Bland–Altman analysis identified differences between the devices, with Vitruve measuring 0.009 to 0.079 m·s^−1^ less than T-Force at moderate and low intensities. Furthermore, the differences revealed a significant bias at moderate (Figure 3E) and low (Figure 3H) intensities, with a tendency for the bias to increase as velocity rose.

### 3.3. Magnitude of Change

The analysis of the magnitude of the differences between the two devices showed trivial effect sizes at high intensity (load) for MPV and PV, small to moderate effect size in MPV at moderate and low intensities, and small effect size in PV at low intensity. Additionally, the differences showed a wide confidence interval for both MPV and PV (~0.7 m/s), mainly at moderate and low loads (Table 1).

## 4. Discussion

The main finding of this study was that Vitruve overestimates MPV (Figure 2) while underestimating PV (Figure 3), mainly at moderate and low loads (at medium and high velocities, respectively). Only one previous study evaluated the validity and concordance of this device at low loads (above 1 m·s^−1^), but using a Smith machine rather than during free-weight exercises [16]. Therefore, the results of the present study could be considered a potentially more practical approach for training [19].

These observed differences could be due to the following: (i) The T-Force device had a sampling rate of 1000 Hz while the Vitruve device operated at 100 Hz. This may have caused the detection of the start and end of the concentric phase to be imprecise in the Vitruve device when determining MPV or may have conditioned the filtering signal to rely on raw data when determining PV [16]. Additionally, it is also possible that the final phase of the movement, which becomes more critical as the bench press velocity increases, was not properly recorded by this device. In other words, measuring with a precision of 100 Hz instead of 1000 Hz could lead to an underestimation of the negative phase of the bench press by Vitruve at high lifting velocities (i.e., overestimating the MPV value), just as it fails to capture the best PV value during the same. (ii) The T-Force device uses a transductor of steel wire while the Vitruve uses a nylon rope, which could be more influenced by friction and heat [22]. This could also explain why Vitruve recorded lower PV and higher MPV compared to T-Force. Over repetitions, the nylon’s elasticity might stretch the rope, reducing rotor torque and lowering PV. Meanwhile, MPV could increase due to the material’s restitution and higher torque during recording.

In the present study, the training load was analysed according to MPV instead of absolute mass or percentage of 1RM, as was the case in previous studies [8,11,15]. Therefore, the practical application might be more transferable to real training, where the users obtain PV or MPV directly from the display of these devices. The MPV has been advocated as a benchmark for determining and monitoring velocity-based strength training [5,16], for the reasons mentioned in the previous paragraph.

Nonetheless, Vitruve may still be a useful tool for monitoring MPV at heavier loads (i.e., slower velocities). These findings (Figure 2) align with those of Kilgallon et al. [15], suggesting that Vitruve is likely valid only at higher intensities (≤0.65 m·s^−1^ for MPV). Similarly, the results are consistent with Callaghan’s study [13], which identified the same limitation in Vitruve when used with lighter loads during free-weight exercises like the bench press. Finally, a recent study found that this device was valid only at MPV < 0.45 m·s^−1^ when measuring the bench press on a Smith machine (~80% of 1RM) [16]. The aforementioned studies consistently reported discrepancies in measuring MPV with the Vitruve device during the bench press exercise at moderate and low loads (i.e., medium and high velocities, respectively). Furthermore, Table 1 shows that the confidence interval for the differences is significantly wider at moderate and low loads than in high ones (~0.7 vs. ~0.4 m/s, respectively). Therefore, the use of this device under these conditions is not recommended.

The main limitations of the present study were as follows: (i) The number of repetitions analysed at low, moderate, and high loads were not uniform (i.e., 65, 62 and 160, respectively), because the study design was performed over an ecological environment of 1RM estimation [19] along a longitudinal training process that required more repetitions at high loads than at moderate and low ones. (ii) Intra-session reliability of the two devices was not measured, because only one repetition at high intensity was available (i.e., only one repetition was performed for each load moved slower than 0.65 m·s^−1^). However, assessing this characteristic was not the aim of the present study, as it has already been analysed in previous research [8,13,15,16]. (iii) The fact of using several measurements from a given participant could cause autocorrelation which overestimates regression statistics [23]. Nonetheless, analysis of correlations was not the focus of this study. (iv) Analysing the data from the two devices (i.e., Vitruve and T-Force) using different software has prevented the isolation of potential errors associated with the software.

## 5. Conclusions

The Vitruve device overestimated MPV and underestimated PV during the free-weight bench press exercise at moderate and low loads (>0.65 m·s^−1^), with these discrepancies becoming more pronounced as the load decreased (i.e., as the velocity increased). These differences were likely caused by the device’s low sampling rate (100 Hz), the processing of the velocity signal (i.e., data analysis), and the components linking to the transducer (e.g., nylon rope). However, it may be a useful tool for monitoring MPV at heavier loads (<0.65 m·s^−1^). Researchers and practitioners should take these findings into account when incorporating the Vitruve into velocity-based strength training protocols.

## Figures and Tables

**Table 1:** Effect size and confidence interval of the differences between T-Force and Vitruve in measuring the mean propulsive velocity (MPV) and the peak velocity (PV) at different intensities (loads) during the bench press exercise.

	MPV		PV	
Loads	*ES*	95% CI	*ES*	95% CI
High	−0.02	−0.24, 0.19	−0.01	−0.23, 0.21
Moderate	−0.26	−0.61, 0.09	0.04	−0.09, 0.60
Low	−0.61	−0.97, −0.25	0.25	−0.31, 0.39

*ES* = effect size; CI = Confidence interval.

## Data Availability

The raw data supporting the conclusions of this article will be made available by consulting the Supplementary materials.

## Supplementary Materials

The following supporting information can be downloaded at: https://www.mdpi.com/article/10.3390/mps8010009/s1. The raw data recorded for each participant (Figure 2 and Figure 3).
